# Study of Ex Vivo and In Vivo Effect of Topical Ionic Solution of Diclofenac Sodium in Male Albino Rats Using Iontophoretic Technique

**DOI:** 10.1155/adpp/7708719

**Published:** 2026-04-30

**Authors:** Hasnan Ali, Sadia Ahmed Zuberi, Noreen Tariq

**Affiliations:** ^1^ Product Lead, Manufacturing Sciences and Technology, GlaxoSmithKline, Karachi, Pakistan, gsk.com; ^2^ Faculty of Pharmacy, Salim Habib University, Karachi, Pakistan, shu.edu.pk; ^3^ Baqai Medical University, Baqai Institute of Pharmaceutical Sciences, Karachi, Pakistan, baqai.edu.pk

**Keywords:** diclofenac sodium, ionic solution, iontophoretic device, transdermal drug delivery

## Abstract

**Background:**

This study is designed to overcome the limitations of transdermal drug delivery by developing a customized iontophoretic device to rapidly increase the delivery of NSAIDs (diclofenac sodium [DS]).

**Methods:**

Three concentrations of DS topical solution were prepared. A customized iontophoretic device has been designed to study the effect of the device in vitro, ex vivo, and in vivo on the release of DS topical solution.

**Results:**

Release of DS using an iontophoretic device was found proportional to the drug concentration with respect to time and current applied, i.e., 2.0% solution showed more promising drug release than 1.5% and 1.0%. Likewise, the release rate of DS is much higher at 5 mA than at 1 mA current. The most prominent in vitro concentration of DS (2%) showed a significant reduction in the rat’s paw size compared with the in vivo control and passive control groups.

**Conclusion:**

The consistency of these results across both ex vivo and in vivo studies underscores the efficacy and preclinical proof‐of‐concept for this iontophoretic technique. While these results are promising, further studies on long‐term skin tolerability and irritation are required to establish its safety profile for human use in enhancing drug delivery. This work provides a solid foundation for further development of noninvasive drug delivery systems for topical applications, offering a promising approach for improving therapeutic outcomes.

## 1. Introduction

Nonsteroidal anti‐inflammatory drugs (NSAIDs) are considered the most popular over‐the‐counter (OTC) medicines. It is widely used for pain, inflammation, and other musculoskeletal conditions. It is estimated that approximately 5%–10% of the prescribed medications per annum are NSAIDs [1, 2]. Besides their associated side effects like GI bleeding, hypertension, and kidney damage, they are still considered a prime class of drugs for treating mild to moderate pain [3, 4]. NSAIDs act as inhibitors of cyclooxygenase enzymes (COX–I and COX–II) and ultimately halt the cascade of inflammation [5–8].

The side effects of NSAIDs are heightened in conventional dosage forms, such as tablets that are used for a prolonged period and at high doses [7]. Diclofenac is one of the most frequently prescribed NSAIDs, used as sodium or potassium salt in various dosage forms like tablets, capsules, injections, modified‐release dosage forms, sprays, and topical preparations [8–11]. Extensive topical use of diclofenac in treating arthritis is preferred, especially in conventional gels and/or creams, which reduce systemic side effects and improve patient compliance. The major drawback is their poor drug permeation, which affects their efficacy [12, 13].

A transdermal drug delivery system (TDDS) is a system applied to the skin for the delivery of medication at a controlled pace into the systemic circulation at clinically sufficient concentrations to assure therapeutic efficacy over a long period [14, 15]. The most significant component of the topical drugs is the skin, which is considered the fundamental element in TDDS. The skin is the largest organ of the body, facilitating the application of a variety of pharmaceutical dosage forms, providing local and systemic effects. However, the leading limitation of TDDS is the penetration of the drug through the stratum corneum [16, 17]. To elude this hindrance and enhance drug flux and penetration through the skin membrane, numerous strategies are designed, comprising chemical enhancers and/or physical procedures like phonophoresis, electroporation, and iontophoresis [13, 15]. Among these, iontophoresis is considered the most suitable, noninvasive, and localized technique [12, 18]. It is defined as the transport of ionic or charged molecules into tissue by passing a direct or periodic electric current through an electrolyte solution. The ionic solution is delivered using an appropriate polarity where the permeation of ionized drug molecules across the biological membrane occurs under the influence of an electrical current [19]. Generally, applying a low‐voltage electric current (0.5 mA/cm^2^) improves the drug transport across the skin because the gold standard for safety is below 0.5 mA/cm^2^, which prevents skin irritation and irreversible damage [13]. This technique is frequently used to apply different anesthetics and NSAIDs [16, 20].

This study has been designed to develop a customized iontophoretic device that can be used for the rapid delivery of diclofenac sodium (DS), which can be effectively used to treat pain and inflammation. Various in vitro, ex vivo, and in vivo parameters have been investigated to confirm the suitability of the developed device for future clinical applications.

## 2. Materials and Methods

### 2.1. Materials

DS was gifted/donated by Barrett Hodgson Pakistan Pvt Ltd. Dimethyl sulfoxide and propylene glycol were purchased from Sigma‐Aldrich, and ethanol was purchased from BDH‐AnalaR. All other solvents/chemicals used in this study were of analytical grade, having the highest degree of purity. Freshly prepared RO and deionized water were used for all preparations.

#### 2.1.1. Animals

A total of 42 male albino Wister rats weighing about 150–200 g were purchased from a local supplier. Before the experiment, the animals were kept in the animal house of Baqai Medical University for 24 h at controlled room temperature (25 ± 2°C), with a 12:12 light/dark schedule. All animals were fed *ad libitum* with a commercial diet pellet and water throughout the experiments. All the study procedures and protocols were conducted after approval from the Ethical Committee of Baqai Medical University, Karachi (Ref: BMU‐IREB/09/2024/015).

### 2.2. Methods

#### 2.2.1. Determination of DS via FTIR Spectrometry

The identity and purity of pure DS were confirmed by FTIR spectrometry. A diamond crystal ATR (Smart iTR) sampling assembly attached to a Nicolet iS5 spectrometer (ThermoFisher Scientific Inc., USA) was used to collect the spectra. A total of 64 scans were performed at a resolution of 4 cm^−1^ in between 4500 and 700 cm^−1^ and processed through the built‐in Omnic software (Version 9).

#### 2.2.2. pH Measurements

The pH and temperature of the topical solutions were measured using a glass electrode and the temperature probe, respectively, attached to the pH meter (Accumet, AR10, Fisher Scientific, USA). Commercially available buffer tablets of pH 4.0 and 7.0 (Merck, Germany) were used to make a 100 mL solution in deionized water. The solution was used to calibrate the instrument at room temperature (25 ± 2°C). The glass electrode was immersed directly in the topical solution for a few seconds to equilibrate. The pH of the topical solution was adjusted to 7 (±0.1) using sodium hydroxide (0.1 M) or HCl (0.1 M) solutions.

#### 2.2.3. Preparation of DS Topical Solutions

The topical solution of DS was prepared by dissolving the weighed amount of the drug in DMSO. The solution was stirred using a magnetic stirrer for 20 min in a stoppered glass container at room temperature (25 ± 2°C) to form a clear colorless solution. The composition of DS topical solutions used is outlined in Table 1. During the stirring, the measured volume of PG was added, and the solution was further stirred for 10 min. Thereafter, ethanol was added in a similar pattern, and the stirring continued for a further 10 min. Water was added in small portions during mixing, and the pH of the solution was adjusted to 7 with 0.1 N HCl/NaOH (if required). Once the pH was adjusted, the solution was mixed thoroughly for 10 more minutes, and the volume was made up with the remaining water. A final stirring for 10 min was performed until the solution temperature reached the room temperature (25 ± 2°C).

**TABLE 1:** Different compositions of DS topical solution.

Ingredients	Percentage (w/v)		
DS	1.0	1.5	2.0
DMSO	20	20	20
PG	20	20	20
Ethanol	20	20	20
Water	39	38.5	38

All topical solutions were prepared under uniform conditions and stored at room temperature (25 ± 2°C) in airtight containers protected from light. These solutions were evaluated periodically for any organoleptic changes.

#### 2.2.4. Validation of Assay Method

The absorbance and spectral measurements were made on a double‐beam UV‐visible spectrophotometer (UV‐1601, Shimadzu, Japan) using 10‐mm path‐length quartz cuvettes. All spectral measurements were made at room temperature (25 ± 2°C) under ambient conditions. The instrument carried out automated calibration of the wavelength scale, whereas the absorbance scale was periodically checked using the following calibration standards [21, 22].

#### 2.2.5. Potassium Dichromate (0.050 g/L) in Sulfuric Acid (0.01 N)

 Wavelength: 257 and 350 nm
 Absorbance: 0.723 and 0.536 (±0.01)

#### 2.2.6. Riboflavin in Acetate Buffer (pH 4.0)

 Wavelength: 444 nm
 A (1%, 1 cm): 328

#### 2.2.7. Assay Method for the Determination of DS

An aliquot of 1 mL was pipetted from the drug solution (1, 1.5, or 2%) and was transferred into a 25‐mL volumetric flask (Pyrex). The freshly prepared phosphate buffer solution (pH 7.4) was added to the flask to the required volume, and the flask was shaken continuously for 10 min. An aliquot of 1 mL was pipetted from the stock solution and made up to 25 mL with phosphate buffer. The solution was filtered using a 0.45‐μm membrane filter. The assay was performed spectrophotometrically according to the method of Bucci et al. with slight modification [23]. The absorbance of the sample was measured at 276 nm using phosphate buffer as a blank for baseline correction. A slight modification was made in the assay method; therefore, the linearity, range, repeatability, and sensitivity of the method were re‐determined in accordance with the International Council for Harmonization (ICH) guidelines [24].

The validation of the UV spectrometric method was performed using a stock solution of DS (10 mg) prepared in 100 mL of phosphate buffer. Appropriate dilutions were made from the stock solution of DS in the concentration range of 5–30 μg/mL, and each experiment was performed in sextet to determine the linearity, accuracy, precision, and sensitivity of the method (ICH, 2005). Similarly, the accuracy and precision were also determined on the test solutions of DS (1.0%–2.0%) after making appropriate dilutions.

#### 2.2.8. Design and Development of an Iontophoretic Device

The device was designed uniquely, and the components were selected based on their intended functions. A comparative analysis of previously reported iontophoresis systems with the proposed custom Arduino device is given in Table 2. However, a typical block diagram and the iontophoretic device are given in Figures 1(a), 1(b), respectively. The general description of the components used in the device, along with their functions, is as follows.

**TABLE 2:** Comparative analysis of iontophoretic delivery systems.

Feature Category	Lab scale/Generic power supplies	Proposed custom Arduino device
Economic accessibility	Moderate: ($100–$300) but requires separate peripherals.	Disruptive: Ultra‐low cost (<$50) using off‐the‐shelf components.
Circuit architecture	Linear or switching DC regulation (constant voltage).	Open‐source microcontroller‐based (PWM/constant current).
Current regulation	Manual voltage dial; prone to “current spikes” as skin resistance drops.	Integrated potentiometer (1–5 mA) with active digital feedback.
Safety mechanisms	None: Risk of skin burns if resistance fluctuates.	Dual‐layer safety: physical emergency button + software buzzer alerts.
Data transparency	External multimeter required for any data.	Real‐time telemetry: Built‐in LCD displaying milli‐Ampere, Volts, and time.
Portability and power	Low (Benchtop); requires an AC outlet.	High: modular power via Li‐ion battery or USB‐C compatibility.
Validation method	Manual external verification needed.	Self‐validating: Integrated ammeter for real‐time dose confirmation.

FIGURE 1(a) A typical device block diagram of an iontophoretic system and (b) iontophoretic device.

**Figure:**
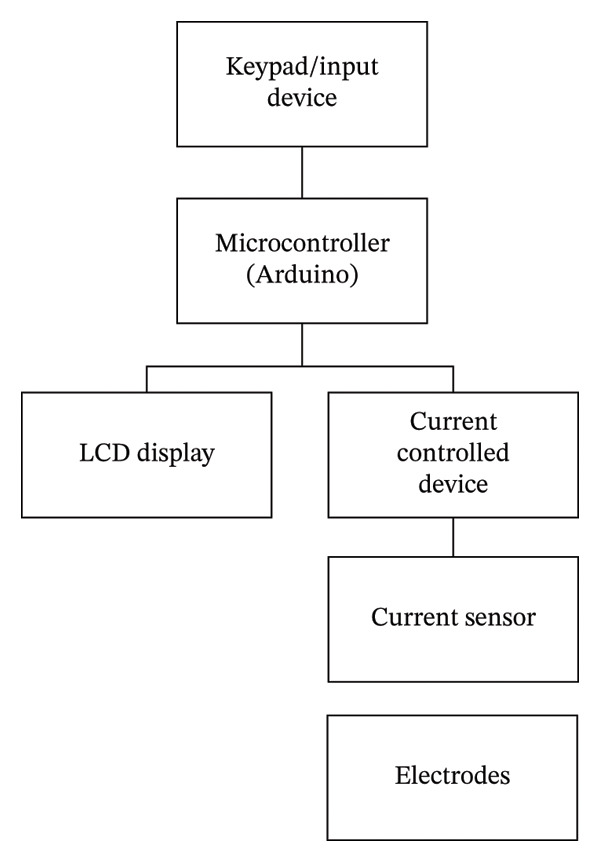
(a)

**Figure:**
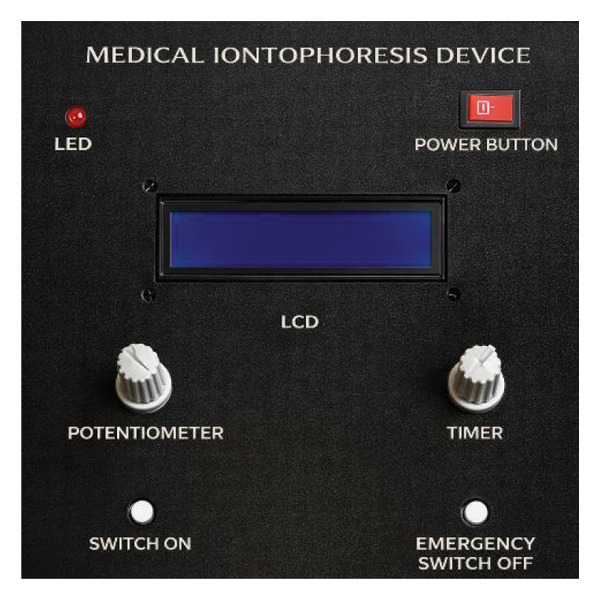
(b)

##### 2.2.8.1. Liquid Crystal Display (LCD)

An LCD is a type of display primarily used to show the device’s operation and the working of other components. A 16 × 2ʺ LCD with an I2C interface was installed with the device.

##### 2.2.8.2. Arduino

It is an open‐source electronics platform based on easy‐to‐use hardware and software. Its function is to read inputs, such as light, a finger on a button, or a Twitter message, and turn them into an output like activating a motor, turning on an LED, or publishing something online. A set of instructions can be sent to the microcontroller on the board. An Arduino programming language (based on Wiring) and the Arduino software (IDE), based on Processing, were used in the device. The current density was kept constant in all experiments.

##### 2.2.8.3. Potentiometer Knob

A potentiometer is a simple knob that provides a variable resistance that can be read into the Arduino board as an analog value. The potentiometer can be used to adjust the voltage (current) as needed.

##### 2.2.8.4. Timer Knob

A timer knob is used to set the time intervals. However, in Arduino, it measures the time interval of an event. An 8‐bit timer was used for the tone function.

##### 2.2.8.5. Arduino Buzzer

A piezo buzzer is a tiny speaker that can be connected directly to an Arduino. Piezoelectricity is an effect where certain crystals change shape when electricity is applied to them. By applying an electric signal at the right frequency, the crystal can make a sound.

##### 2.2.8.6. LED

The LED turns on during circuit operation and turns off when the circuit is not working.

##### 2.2.8.7. Power and Emergency Buttons

A power button was installed on the LCD for activation and deactivation of the device. An emergency switch‐off button was also installed and connected to the Arduino circuit to stop the operation in case of an emergency.

##### 2.2.8.8. Operation Push Button (On/Off Switch)

An operation switch‐on button was installed and connected to the Arduino circuit, and was particularly used for device operation. Once the operating parameters were set via the potentiometer and timer, the button was pressed to start the device operation.

##### 2.2.8.9. Ammeter (Ampere Meter)

An ammeter is an instrument used to measure current in a circuit on both the positive and negative terminals. An ammeter (MT87, Digital Multimeter, China) was used to check that the direct current runs properly throughout the circuit for safety. Checkpoints were set on 1.0, 2.0, 3.0, 4.0, and 5.0 mA to evaluate the receiving current by connecting the terminal(s) in the ammeter for calibration of the device. The iontophoretic device was designed to deliver currents in the range of 1–5 mA. The current fluctuation is around 1–5 mA ± 0.2 mA (0.02 SD) at each set‐point. Depending on electrode‐skin resistance (typically 1–10 kΩ), the corresponding voltage requirement ranged between approximately 5 and 50 V. The circuit automatically adjusted the voltage to maintain the set current, which was manually selected to check drug delivery at different intervals. Overall, the voltage remains stable up to 5 min. The current density was kept constant in all experiments. All experiments were performed in six replicates to assess the reproducibility of the results.

##### 2.2.8.10. Timer

To recheck the time shown on the LCD, a stopwatch was used to calibrate the timer’s functioning. Various checkpoints were set, i.e., 30, 60, 90, 120, and 150 s, to ascertain the proper functioning of the device.

#### 2.2.9. Release Studies of DS

##### 2.2.9.1. In Vitro/Ex Vivo (Active and Passive) Permeation Study With and Without Ionophoresis

The best equivalent of human skin in experimental studies is the rodent’s skin, i.e., whole human skin is 2.97 mm thick, epidermal is 46.9 μm, and stratum corneum is 16.8 μm, while rat’s skin is 0.92–2.80 mm thick, epidermal is 30.4–61.1 μm, and stratum corneum is 13.7–34.7 μm; it shows resemblance between human and rat’s skin [25]. Also, it is a convenient and widely applicable method to use rat skin to conduct permeation studies [26]. The number of animals used in the ex vivo study was 12, i.e., for the ex vivo passive study (without iontophoresis): *n* = 6 and for the ex vivo iontophoresis study (with iontophoresis): *n* = 6.

##### 2.2.9.2. Skin Preparation

Animals were sacrificed by cervical dislocation and then underwent the process of hair removal. Hair removal was followed by applying disinfectant to avoid contamination. Hairs of the abdominal region of the rat were removed by applying depilatory cream (Veet). It was applied for one‐fourth of the manufacturer’s instructions time, i.e., for human use. The maximum time applied was 2 min, after which the hairs were easily removed, and the cream was wiped off the rat’s body. The application time of the depilatory cream is an essential factor to avoid any chemical burns on animal skin [27, 28].

##### 2.2.9.3. Skin Excision

After successfully removing the hair from the rat’s abdominal region, the skin was excised carefully by separating the underlying tissues and fatty debris.

##### 2.2.9.4. Skin Soaking

The excised rat skin was soaked in a phosphate buffer medium (pH 7.4) for 60 min at room temperature before mounting on Franz’s diffusion cell.

##### 2.2.9.5. Skin Mounting on Franz’s Diffusion Cell Apparatus

The rat’s skin was mounted on Franz’s diffusion cell apparatus after soaking the skin in phosphate buffer (pH 7.4).

##### 2.2.9.6. Franz Diffusion Apparatus

The release of DS was studied using a digital 6‐cell diffusion apparatus (Franz diffusion system) attached with a thermostat water circulation tank (Model No. EMFDC 06, Orchid Scientific, India). The body of each cell was filled with 4 ± 0.2 mL of phosphate buffer (pH 7.4), and the receptor solution was stirred with the help of a magnetic bead at a speed of 100 rpm at 32 ± 0.5°C. The rodent’s skin was used in place of the membrane. The rodent’s skin was soaked for 30 min in the receptor medium before use and then placed between the donor chamber and the receptor chamber of the Franz diffusion cell and fixed with the help of aluminum clips.

One mL of DS topical solution was added to the donor chamber using a syringe. The negative terminal (cathode) of the device was placed in the donor compartment using copper wire to enhance the flux of the anionic drug; the positive terminal (anode) of the device was placed in the sampling port of the cell using copper wire to get the deposition of free ions. The release of the drug was calculated using the following formula:

(1) % drug release=absorbance of the sampleabsorbance of the standard×100.

##### 2.2.9.7. Release Kinetics

The release kinetics of DS from each compounded solution have been determined using various equations, including zero‐order, first‐order, and Higuchi model by using the following formulas (Helal et al., 2012; Higuchi, 1961, 1963; Shoaib et al., 2010):

(2) zero−order: Qt=k0t,

where *Q*_*t*_ is the amount of drug release in time *t*; *k*_0_ is the zero‐order rate constant expressed in units of concentration/time, and *t* is the time in *h*:

(3) first−order:Log Q=LogQ0 – kt2.303,

where *Q*_0_ is the initial concentration of the drug, *k* is the first‐order rate constant, and *t* is the time in *h*:

(4) Higuchi model: Q=kH t,

where *Q* is the amount of drug release, *k*_*H*_ is the release rate constant, and *t* is the time in *h*.

##### 2.2.9.8. Standard Operating Procedure of the Iontophoretic Device Connected With the Franz Diffusion Apparatus

Following is the brief step‐wise procedure of operating the medical iontophoresis device used in this study for the release of DS from the Franz diffusion apparatus:

i. Turn on the device from the “Main Power Button” by connecting the USB port with the “Power Bank.”
ii. Set the current (in mA) from the potentiometer knob and time (in seconds) from the timer knob by getting a display on the LCD.
iii. After setting both the parameters (i.e., current and time), press the “operation push button” to start the delivery of the drug in the cell across the skin membrane.
iv. The samples were withdrawn at different times (0, 1, 2, 3, 4, and 5 min) and current (1, 2, 3, 4, and 5 mA) intervals.
v. A quantity of 1 mL was withdrawn from the sampling port and transferred to a 5‐mL volumetric flask, and made up to volume with the phosphate buffer. The assay using this solution was performed similarly to that discussed in Section “Assay of DS.”
vi. Each time an equal amount of fresh receptor medium was added to maintain the sink condition.
vii. Once the set time was finished, the delivery of the drug and the operation of the device stopped automatically.

NOTE: In case of an emergency during operation, the “Emergency Push Button” is present to stop the functions immediately.

##### 2.2.9.9. In Vivo Assay

###### 2.2.9.9.1. Carrageenan‐Induced Paw Edema in Rats

To determine the anti‐inflammatory activity of DS, edema was induced by injecting 0.1 mL of 1% freshly prepared carrageenan in normal saline at the subplantar region of the right hind paw of the rat. It is a precise and widely applicable method for evaluating the anti‐inflammatory effect of drugs [29, 30]. The rat’s paw size was measured before and after carrageenan injection with the help of a Vernier caliper, and paw volume was measured with the help of a plethysmometer at 60, 120, and 180 min to estimate the anti‐inflammatory effect of DS.

###### 2.2.9.9.2. Experimental Protocol

To analyze the anti‐inflammatory activity of DS with the iontophoresis technique, the same doses of samples on which ex vivo studies have been done were used. The animals were divided randomly into three groups (*n* = 10 per group). The details are as follows.

###### 2.2.9.9.3. Group # 1: Control

Inflammation was induced in rats, and the paw size and volume were measured as mentioned above.

###### 2.2.9.9.4. Group # 2: Passive Control

Animals were anesthetized by ketamine and xylazine at the ratio of 1:2, i.e., K:100 mg/kg and X:10 mg/kg, by intraperitoneal administration [31]. Afterward, a topical solution of DS was applied to the abdominal region of rats. After 1.5 h, 0.1 mL of 1% carrageenan freshly prepared in normal saline was injected at the subplantar region of the right hind paw of the rat, and the paw size and volume were measured at different time intervals as mentioned above.

###### 2.2.9.9.5. Group # 3: Iontophoresis Treatment

The skin was prepared as explained previously. Afterward, a topical solution of DS was applied to the abdominal region of rats along with the iontophoresis treatment at a current intensity of 1–5 mA. After 1.5 h, 0.1 mL of 1% freshly prepared carrageenan in normal saline was injected into the subplantar region of the right hind paw of the rat, and the paw size and volume were measured as explained above.

###### 2.2.9.9.6. Statistical Analysis

One‐way ANOVA was used to analyze the data, followed by Dunnett’s multiple comparisons (SPSS 21). The *p*‐value of < 0.05 was considered significant.

## 3. Results and Discussion

### 3.1. Confirmation of DS via FTIR Spectrometry

**FIGURE 2:**
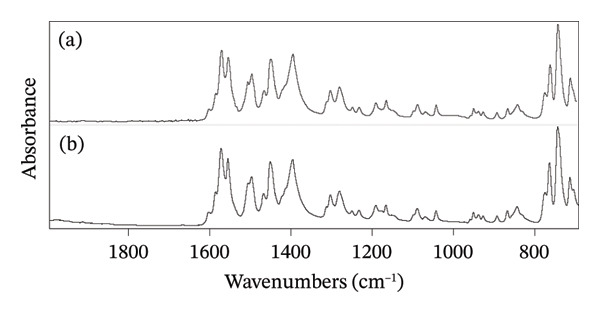
FTIR spectra of DS (a) sample and (b) reference standard.

The identity and purity of DS have been confirmed through FTIR spectrometry. The sample powder has been compared with the reference standard of DS (Figure 2). The major peaks observed at 1572 and 1556 cm^−1^ correspond to the carboxylate group stretching, i.e., C=O and C=C, respectively. The bending peaks at 1451 and 1397 cm^−1^ are for the CH_3_ group, while the C–N stretching peaks were observed at 1305 and 1282 cm^−1^. A prominent peak has been observed at 745 cm^−1,^ corresponding to C–Cl stretching. All principal peaks observed correspond to the reported values, and no additional peaks have been identified, indicating that the material is pure [23, 32–36].

### 3.2. pH of the Topical Solutions of DS

It has been observed that lowering the pH below 5.5 results in precipitation, leading to instability of the topical DS solution. Such instability has also been observed by Bucci et al. [23]. Therefore, the pH of all topical solutions has been set to 7.0 (±0.1), which is within the optimum range for DS stability. The solubility of DS increases with pH at values exceeding the *p*K_a_ (*p*K_a_ 4) [37]. At physiological pH, DS exists as ions, which makes it an ideal candidate for cathodal iontophoresis.

### 3.3. Preparation of DS Topical Solutions

During the preparation of the DS topical solution, an exothermic reaction has been observed. The temperature of the topical solution was measured with a digital thermometer, which initially showed above 45°C. When this solution was subjected to UV spectrometric analysis, an inappropriate, nonreproducible, and considerably high absorbance (> 1.000) was noted. On the contrary, when the topical solution was prepared in a cooling water bath or kept at room temperature for a few minutes, the solutions showed appropriate and reproducible absorbance each time. Therefore, it is important to bring the temperature of the topical solution to normal (below 30°C) before the spectrometric analysis.

### 3.4. Validation of Assay Method

**FIGURE 3:**
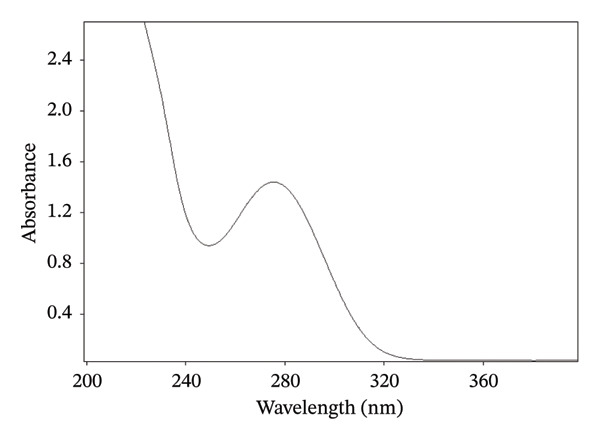
UV spectrum of DS in phosphate buffer at pH 7.4.

The DS assay was performed according to the method reported by Bucci et al., after slight modification [23]. In the present study, a phosphate buffer of pH 7.4 has been used instead of deionized water and the absorbance has been measured at 276 nm (Figure 3). Therefore, to ascertain the assay results, the linearity, accuracy, precision, and sensitivity of the test method have been determined as per the guidelines of ICH (2005) and the results are reported in Table 3 [24].

**TABLE 3:** Validation data^a^ for the analysis of DS by the UV spectrometric method.

*λ*_max_	276 nm
Linearity Range Correlation coefficient Slope Intercept SE^b^ of slope SE^b^ of intercept SD^c^ of intercept	5–30 μg/mL 0.9999 0.03 1.66 × 10^−2^ 5.47 × 10^−3^ 0.005 1.25 × 10^−2^

Recovery range (%)^d^	98.08–101.33
Accuracy (%)^e^ ± SD^c^	99.79 ± 1.049
Precision (%RSD)^f^	1.051
LOD^g^ (μg/mL)	1.23
LOQ^h^ (μg/mL)	3.72

^a^ *n* = 6.

^b^SE = standard error.

^c^SD = standard deviation.

^d^Recovery (%) = (amount found/amount added) × 100.

^e^Accuracy (%) = mean recovery range.

^f^%Relative standard deviation =  (SD/mean) × 100.

^g^Limit of detection = 3.3 × (SD of intercept/slope).

^h^Limit of quantification = 10 × (SD of intercept/slope).

**FIGURE 4:**
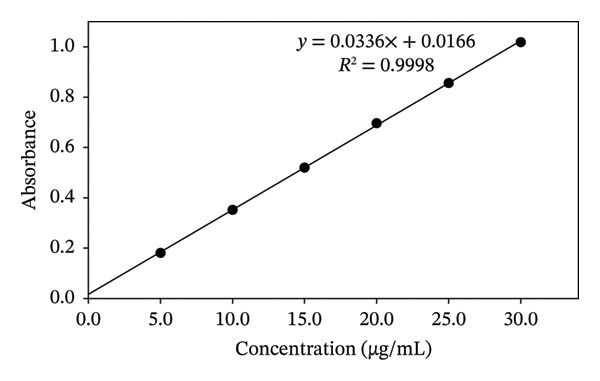
Calibration curve for the assay of DS.

**FIGURE 5:**
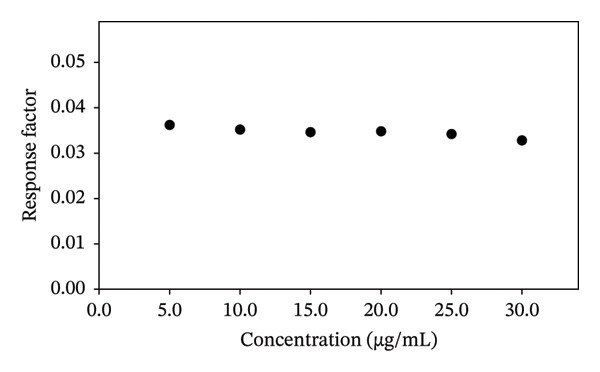
A plot of response factor versus concentration of DS.

The test method has been found linear in the concentration range of 5–30 μg/mL with an *R* ^2^ value of 0.9998 (Figure 4). The response factor (RF) has also been calculated by dividing the mean concentration by the theoretical concentration to confirm the linearity range results of the assay method (Figure 5). The values suggested that the studied concentration range is linear within the studied conditions and parameters of the assay method, as the ratio obtained was nearly the same for all concentrations, i.e., ∼0.03 (Figure 5). The method is found to be highly accurate (99.79 ± 1.049%) and precise (1.051%), and the recovery range is found to be between 98.08% and 101.33% (Table 3). Moreover, the method is highly sensitive, as indicated by the limit of detection (LOD) and limit of quantification (LOQ) values, confirming that the test method is suitable for DS assay (Table 3).

### 3.5. Assay of DS

The developed assay method has been applied to the compounded extemporaneous topical solutions for the DS quantification. The assay results are found to be accurate and precise as reported in Table 4.

**TABLE 4:** Drug recovery from the prepared topical solutions by the applied UV spectrometric method (*n* = 6).

DS (%)	Mean drug recovery (%) ± SD	%RSD
1.0	100.11 ± 1.02	1.02
1.5	101.47 ± 1.08	1.06
2.0	102.05 ± 1.54	1.51

### 3.6. Release Studies of DS

#### 3.6.1. In Vitro/Ex Vivo (Active) Permeation Study With Iontophoresis Versus Without Iontophoresis

**FIGURE 6:**
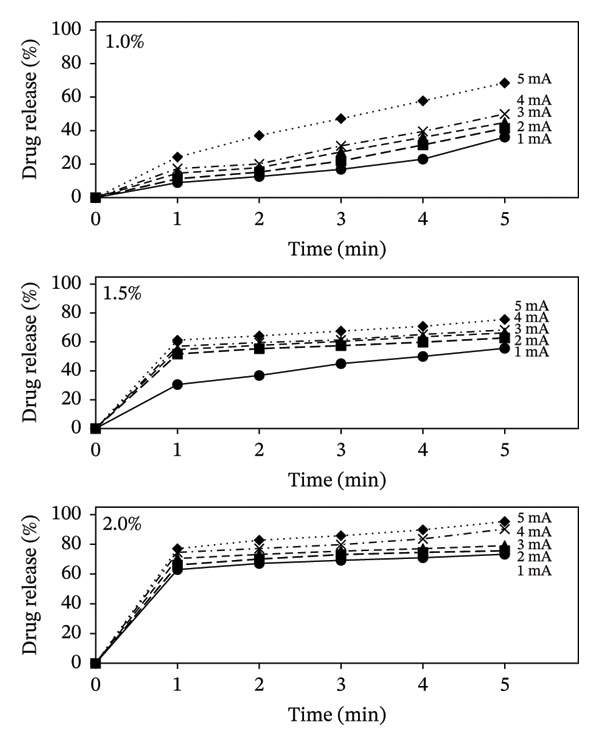
Release of DS with ionophoresis from the topical solutions (1%–2%) at different current and time intervals (*n* = 6).

The release of DS has been studied using a digital 6‐cell diffusion apparatus (Franz diffusion system) connected with the iontophoretic system. The device was connected to the Franz diffusion apparatus to mimic the procedure of drug delivery using a patch, i.e., applied to the human skin. The effect of current on drug release has been studied with respect to time and drug concentration (Figure 6). An increase in drug release with time has been observed with increasing current (Table 5). The data are compared with the passive release of DS topical solution without ionophoresis (Table 6). This indicates that the release of the ionized drug is directly proportional to the amount of current delivered (Figure 6). The higher drug release could be due to increased polarization between the solution and ions.

**TABLE 5:** Cumulative drug release from the topical solutions of DS (1.0%–2.0%) at different times and current intervals with ionophoresis (*n* = 6).

Time (min)	1.0% DS topical solution Cumulative drug release (%) ± SD				
	1 mA	2 mA	3 mA	4 mA	5 mA
1	8.95 ± 10.63	11.25 ± 12.28	14.60 ± ±12.53	17.26 ± ±13.59	24.20 ± ±17.28
2	12.53 ± 10.63	15.22 ± 12.28	17.90 ± 12.53	20.02 ± 13.59	37.10 ± 17.28
3	16.84 ± 10.63	21.85 ± 12.28	27.20 ± 12.53	30.91 ± 13.59	47.10 ± 17.28
4	22.89 ± 10.63	31.42 ± 12.28	36.00 ± 12.53	39.52 ± 13.59	57.70 ± 17.28
5	36.05 ± 10.63	41.43 ± 12.28	44.80 ± 12.53	49.92 ± 13.59	68.50 ± 17.28

**Time (min)**	**1.5% DS topical solution** **Cumulative drug release (%) ± SD**				
	**1 mA**	**2 mA**	**3 mA**	**4 mA**	**5 mA**

1	30.51 ± 10.07	51.57 ± 4.25	54.72 ± 4.58	56.98 ± 4.50	61.19 ± 5.61
2	36.71 ± 10.07	55.38 ± 4.25	57.59 ± 4.58	59.49 ± 4.50	64.19 ± 5.61
3	45.00 ± 10.07	57.40 ± 4.25	60.29 ± 4.58	61.40 ± 4.50	67.54 ± 5.61
4	50.00 ± 10.07	59.83 ± 4.25	63.55 ± 4.58	65.15 ± 4.50	70.77 ± 5.61
5	55.59 ± 10.07	62.73 ± 4.25	66.22 ± 4.58	68.30 ± 4.50	75.57 ± 5.61

**Time (min)**	**2.0% DS topical solution** **Cumulative drug release (%) ± SD**				
	**1 mA**	**2 mA**	**3 mA**	**4 mA**	**5 mA**

1	63.05 ± 3.91	66.09 ± 3.85	70.42 ± 3.36	74.52 ± 6.15	77.02 ± 6.91
2	67.13 ± 3.91	70.12 ± 3.85	73.23 ± 3.36	77.15 ± 6.15	82.76 ± 6.91
3	69.26 ± 3.91	73.06 ± 3.85	75.43 ± 3.36	79.75 ± 6.15	85.78 ± 6.91
4	70.91 ± 3.91	74.55 ± 3.85	77.06 ± 3.36	83.60 ± 6.15	89.68 ± 6.91
5	73.34 ± 3.91	75.65 ± 3.85	79.09 ± 3.36	90.28 ± 6.15	95.30 ± 6.91

**TABLE 6:** Ex vivo (passive) cumulative drug release without ionophoresis from the topical solutions of DS (1.0%–2.0%) at different times (*n* = 6).

Time (min)	1.0% DS topical solution Cumulative drug release (%) ± SD
1	42.49 ± 0.28
2	42.85 ± 0.28
3	42.96 ± 0.28
4	43.01 ± 0.28
5	43.25 ± 0.28

**Time (min)**	**1.5% DS topical solution** **Cumulative drug release (%)** **± SD**

1	48.68 ± 0.17
2	48.75 ± 0.17
3	48.81 ± 0.17
4	48.87 ± 0.17
5	49.12 ± 0.17

**Time (min)**	**2.0% DS topical Solution** **Cumulative drug release (%)** **± SD**

1	69.62 ± 0.13
2	69.75 ± 0.13
3	69.83 ± 0.13
4	69.89 ± 0.13
5	69.97 ± 0.13

**FIGURE 7:**
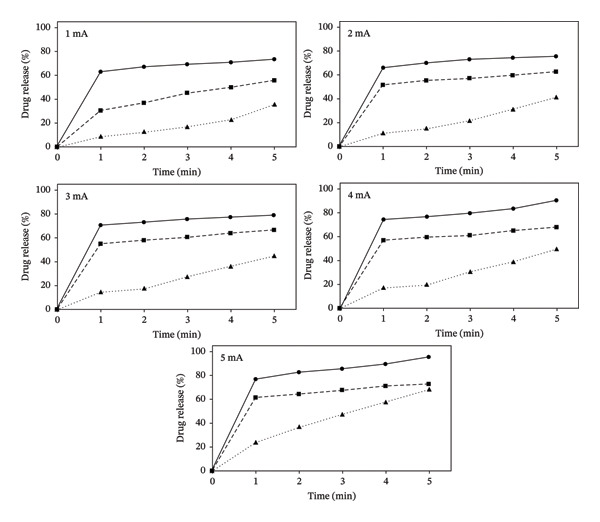
Effect of current (1–5 mA) on the release of DS (1%–2%) at different time intervals (*n* = 6).

Similarly, it has also been observed that the drug release increases with an increase in the concentration of the drug with respect to time and current (Figure 7). Hence, the more the ionized drug molecules present in the solution, the higher will be the release of the drug from the system. This could probably be due to the increase in the concentration of ionized drug in the solution. This difference in the drug release is more prominent between the solutions of 1%–1.5% and 2.0% as compared to 1.5% and 2.0% (Figure 7). On the contrary, a much‐decreased drug release has been observed in DS topical solutions at all concentrations without ionophoresis.

A significant increase in DS drug release was observed with the iontophoretic device, i.e., 95% drug was released in 2.0% DS topical solution (Table 5). In contrast, 69% of the drug release was observed without current in 2.0% DS topical solution (Table 6).

The release kinetics of DS from different concentrations (1.0%–2.0%) of topical solution at different currents (1–5 mA) and time intervals (1–5 min) with ionophoresis have been investigated by using zero‐order, first‐order, and Higuchi model equations (Table 7).

**TABLE 7:** Data on the release kinetics of DS solution (1.0%–2.0%) with ionophoresis

Current (mA)	1.0% DS topical solution					
	Zero order		First order		Higuchi model	
	*R* ^2^	*k*_0_ (*h* ^−1^)	*R* ^2^	*k* (*h* ^−1^)	*R* ^2^	*k*_0_ (*h* ^−1^)
1	0.954	6.47	0.927	0.08	0.851	14.09
2	0.984	7.84	0.969	0.10	0.899	17.27
3	0.983	8.50	0.982	0.11	0.936	19.13
4	0.977	9.35	0.976	0.13	0.939	21.15
5	0.968	12.94	0.993	0.22	0.985	30.12

**Current (mA)**	**1.5% DS topical solution**					
	**Zero order**		**First order**		**Higuchi model**	
	**R** ^2^	**k_0_(h^−1^)**	**R** ^2^	***k* (h^−1^)**	**R** ^2^	**k_0_(h^−1^)**

1	0.854	9.85	0.930	0.15	0.989	24.44
2	0.588	9.73	0.673	0.16	0.844	26.88
3	0.592	10.29	0.690	0.18	0.846	28.37
4	0.584	10.51	0.685	0.18	0.838	29.05
5	0.606	11.71	0.744	0.23	0.854	32.07

**Current (mA)**	**2.0% DS topical solution**					
	**Zero order**		**First order**		**Higuchi model**	
	**R** ^2^	**k**_0_ (h^−1^)	**R** ^2^	***k* (h^−1^)**	**R** ^2^	**k_0_(h^−1^)**

1	0.550	11.21	0.651	0.21	0.816	31.50
2	0.541	11.62	0.644	0.23	0.810	32.79
3	0.526	11.93	0.634	0.25	0.796	33.86
4	0.588	13.75	0.808	0.37	0.839	37.88
5	0.601	14.79	0.885	0.51	0.852	40.62

All three equations were applied, and it has been observed that there is a concentration effect on the release of DS from the compounded solutions (Table 7). In the case of 1.0% solution, the data best fit the first‐order release equation, while in higher concentration solutions (1.5% and 2.0%), the release of DS followed the Higuchi model (Table 6). This indicates that at lower concentrations, the release of DS is proportional to the log concentration versus time, while at the higher concentrations, the cumulative percent drug release becomes proportional to the square root of time. In the Higuchi model, the drug is released in a time‐dependent manner, and the results of this study (Table 5, Figs. 6 and 7) have shown that an increase in the concentration of the drug resulted in higher release of DS (Table 7).

#### 3.6.2. In Vivo Drug Release

The concentration of DS that showed the most prominent in vitro results (i.e., 2%) was selected for in vivo studies. The inflammation of the rat’s paw reaches a maximum in 1 h and declines within 3 h. The passive group that received the topical solution of DS showed a reduction in inflammation as compared to the control group, i.e., 33.33% (*p*‐value < 0.05), while the group of animals that received DS 2% along with iontophoresis at 5 mA for 3 min showed a significant reduction in paw size, i.e., 62.19% (*p*‐value < 0.05) in comparison with control and passive control groups (Table 8). However, the percentage inhibition of DS with and without iontophoresis in the carrageenan‐induced paw edema model of rats is given in Table 9.

**TABLE 8:** Effect of different treatments on the volume of carrageenan‐induced paw edema in the rat model.

Group	PAW edema volume of rats		
	60 min	120 min	180 min
Control	0.765 mL	0.87 mL	0.96 mL
Passive control	0.623 mL	0.713 mL	0.64 mL
DS (2%) iontophoresis	0.446 mL	0.536 mL	0.363 mL

**TABLE 9:** Evaluation of the percentage inhibition of DS in the carrageenan‐induced paw edema model of rats.

Group	Percentage inhibition		
	60 min	120 min	180 min
Passive control	18.63%^∗^	17.99%^∗∗^	33.3%^∗^
DS (2%) iontophoresis	41.69%^∗^	38.39%^∗∗^	62.19%^∗∗^

*Note:* The asterisks (^∗^) represent the significant difference between the DS‐only group and the control normal, and the DS with iontophoresis‐treated group (^∗^ *p* < 0.05, ^∗∗^ *p* ∼ 0.001).

**FIGURE 8:**
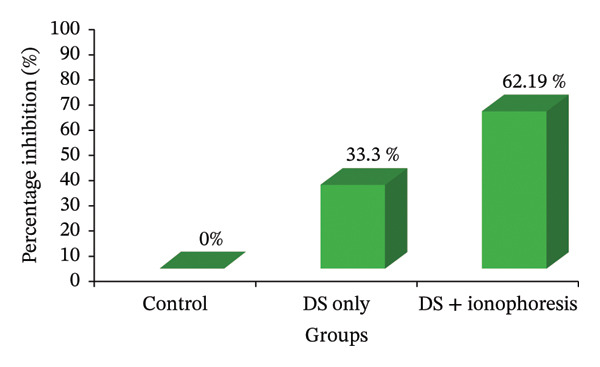
Analysis of anti‐inflammatory activity of DS with and without iontophoresis.

Figure 8 represents the anti‐inflammatory activity as a reduction in paw volume by the treatment of DS with and without iontophoresis in the Carrageenan‐induced paw edema model of rats. In this graph, each bar shows mean ± SEM (*n* = 6). For statistical analysis, one‐way ANOVA was applied, i.e., followed by Dunnett’s multiple comparisons (SPSS 21).

The consistency of these results across both ex vivo and in vivo studies underscores the efficacy and potential preclinical significance of this iontophoretic technique in enhancing drug delivery. This work establishes a compelling proof‐of‐concept for advancing noninvasive drug delivery systems for topical applications, offering a promising pathway toward improved therapeutic outcomes and laying the groundwork for future translational research.

#### 3.6.3. Correlation Between Ex Vivo Permeation and In Vivo Efficacy

To understand the relationship between the extent of DS delivered and the therapeutic efficacy, correlation analysis was studied between the cumulative ex vivo drug permeation and the percentage inhibition of paw edema. A strong linear correlation was observed (Table 10), suggesting that the improvement in drug permeation with the iontophoretic delivery system at 5 mA was responsible for the reduction in inflammation (62.19%). The correlation analysis confirms that the Franz diffusion model is a predictive tool for the anti‐inflammatory activity of the iontophoretic system in vivo.

**TABLE 10:** Correlation between ex vivo permeation and in vivo efficacy.

Group	Ex vivo permeation (at 5 min)	In vivo % inhibition
Passive (no current)	Low	33.33%
Iontophoretic (1 mA)	Medium–low	∼40% (Estimated)
Iontophoretic (5 mA)	High	62.19%

## 4. Conclusion

This study successfully evaluated the ex vivo and in vivo effects of a topical ionic solution of DS in male albino rats using a custom‐designed portable iontophoretic system. The release profile of DS was assessed using the Franz diffusion apparatus at varying concentrations, current intensities, and time intervals. The findings demonstrate that a 2% DS solution consistently yielded superior drug release compared to 1.5% and 1% solutions. Specifically, application of 5 mA current for 5 min facilitated approximately 95% drug release from the 2% solution, establishing it as the optimal formulation and parameter for effective delivery.

The consistency of these results across both ex vivo and in vivo studies underscores the efficacy and preclinical proof‐of‐concept for this iontophoretic technique. While these results are promising, further studies on long‐term skin tolerability and irritation are required to establish its safety profile for human use in enhancing drug delivery. This work provides a solid foundation for further development of noninvasive drug delivery systems for topical applications in humans, offering a promising approach for enhanced localized drug delivery for future translational research.

## Funding

No funding was received for this manuscript.

## Conflicts of Interest

The authors declare no conflicts of interest.

## Data Availability

The data that support the findings of this study are available from the corresponding author upon reasonable request.
